# Effects of Increased Von Willebrand Factor Levels on Primary Hemostasis in Thrombocytopenic Patients with Liver Cirrhosis

**DOI:** 10.1371/journal.pone.0112583

**Published:** 2014-11-14

**Authors:** Andreas Wannhoff, Oliver J. Müller, Kilian Friedrich, Christian Rupp, Petra Klöters-Plachky, Yvonne Leopold, Maik Brune, Mirja Senner, Karl-Heinz Weiss, Wolfgang Stremmel, Peter Schemmer, Hugo A. Katus, Daniel N. Gotthardt

**Affiliations:** 1 Department of Internal Medicine IV, University Hospital Heidelberg, Heidelberg, Germany; 2 Department of Internal Medicine III, University Hospital Heidelberg, Heidelberg, Germany; 3 Department of Internal Medicine I and Clinical Chemistry, University Hospital Heidelberg, Heidelberg, Germany; 4 Department of General and Transplant Surgery, University Hospital Heidelberg, Heidelberg, Germany; University of Pisa, Italy

## Abstract

In patients with liver cirrhosis procoagulant and anticoagulant changes occur simultaneously. During primary hemostasis, platelets adhere to subendothelial structures, via von Willebrand factor (vWF). We aimed to investigate the influence of vWF on primary hemostasis in patients with liver cirrhosis. Therefore we assessed in-vitro bleeding time as marker of primary hemostasis in cirrhotic patients, measuring the Platelet Function Analyzer (PFA-100) closure times with collagen and epinephrine (Col-Epi, upper limit of normal ≤165 s) or collagen and ADP (Col-ADP, upper limit of normal ≤118 s). If Col-Epi and Col-ADP were prolonged, the PFA-100 was considered to be pathological. Effects of vWF on primary hemostasis in thrombocytopenic patients were analyzed and plasma vWF levels were modified by adding recombinant vWF or anti-vWF antibody. Of the 72 included cirrhotic patients, 32 (44.4%) showed a pathological result for the PFA-100. They had mean closure times (± SD) of 180±62 s with Col-Epi and 160±70 s with Col-ADP. Multivariate analysis revealed that hematocrit (*P* = 0.027) and vWF-antigen levels (*P* = 0.010) are the predictors of a pathological PFA-100 test in cirrhotic patients. In 21.4% of cirrhotic patients with platelet count ≥150/nL and hematocrit ≥27.0%, pathological PFA-100 results were found. In thrombocytopenic (<150/nL) patients with cirrhosis, normal PFA-100 results were associated with higher vWF-antigen levels (462.3±235.9% vs. 338.7±151.6%, *P* = 0.021). These results were confirmed by multivariate analysis in these patients as well as by adding recombinant vWF or polyclonal anti-vWF antibody that significantly shortened or prolonged closure times, respectively. In conclusion, primary hemostasis is impaired in cirrhotic patients. The effect of reduced platelet count in cirrhotic patients can at least be partly compensated by increased vWF levels. Recombinant vWF could be an alternative to platelet transfusions in the future.

## Introduction

Patients with liver cirrhosis suffer from complex abnormalities of the hemostatic system that affect primary and secondary hemostasis and fibrinolysis. Changes in procoagulant and anticoagulant proteins occur simultaneously [1], [2], and yet it has not been understood if an individual patient currently is in favor of bleeding or thrombosis. Thus, these patients can clinically present with an increased bleeding risk or an increased rate of venous thrombosis as well as develop portal vein thrombosis [3], [4].

During primary hemostasis, platelets adhere to the subendothelium via the von Willebrand factor (vWF) [5]. Thrombocytopenia, thrombocytopathy, or impaired function of vWF can disrupt primary hemostasis. Thrombocytopenia is common in liver cirrhosis, occurring in up to 70% of patients even though the underlying pathomechanisms are most likely multifactorial and not yet fully understood [6]; it is assumed that an imbalance between the production and survival of platelets is its most likely cause. Thrombocytopenia can be due to bone marrow suppression or decreased thrombopoietin levels. Hypersplenism, platelet consumption, and platelet autoantibody action might decrease survival of platelets in cirrhotic patients [7]. Data on concomitant thrombocytopathy is not clear and in part controversial. As an example of thrombocytopathy, impaired platelet activation has been reported, while other results indicate hyperactivation of platelets in end-stage liver disease [7]. Levels of vWF are elevated in cirrhosis, which may be due to different causes. Endotoxemia in patients with liver cirrhosis has shown to increase vWF-levels [8]. Further, vWF mRNA levels in hepatic tissue are increased as well, indicating increased hepatic synthesis of vWF [9].

In hemostasis, von Willebrand factor binds to glycoprotein Ib on the platelet surface and to the subendothelial matrix. Besides this, vWF was recently recognized as playing important roles during angiogenesis, inflammation, cell proliferation and tumor cell growth [10]. Further, vWF levels were shown to correlate with the degree of portal hypertension as measured by hepatic venous pressure gradient and vWF levels were predictive of survival free of portal hypertension-related events and liver transplantation [11]. They were as well associated with development of hepatopulmonary syndrome [12] and in a further study prediction of mortality in patients with end-stage liver disease was equal to MELD score [13]. vWF is cleaved by the protease ADAMTS13 (a disintegrin-like and metalloprotease with thrombospondin type 1 motif 13), which is mainly synthesized in the liver and found to be reduced in cirrhosis [14], [15].

Measurement of the in-vitro bleeding time using the Platelet Function Analyzer (PFA-100) is an established method for assessing primary hemostasis. It shows almost no intra-assay variability and has a high accuracy for detection of platelet function defects with an area under the ROC curve of 0.977 [16]. It successfully aids detection of patients with platelet function defects or von Willebrand's disease [17].

We assessed the PFA-100, a test that closely represents the in-vivo setting, in cirrhotic patients to investigate primary hemostasis. Therefore, we aimed to study the effect of increased levels of vWF on the in-vitro bleeding time in thrombocytopenic patients.

## Materials and Methods

### Patients

Patients with end-stage liver disease on the waiting list for liver transplantation at the Heidelberg University Hospital (Germany) were eligible for inclusion, regardless of etiology, platelet count, or hematocrit levels. Patients who had taken either antiplatelet drugs, non-steroidal anti-inflammatory drugs (NSAID), or other anticoagulant agents within the last ten days were excluded; patients were also excluded if no information on these drugs was available.

We measured a complete blood count as well as PFA-100 closure-times after stimulation with epinephrine or ADP, vWF-antigen, and vWF-activity as well as the ratio vWF-activity:vWF-antigen and ADAMTS13 activitiy. Analyses were separately conducted for the whole study cohort, for patients without thrombocytopenia and without anemia (defined as platelet count ≥150/nL and hematocrit ≥27%) and for patients with thrombocytopenia (<150/nL). Analysis was as well performed for patients with a platelet count <60/nL, which is an accepted cut-off for performing invasive procedures, such as transcutaneous liver biopsy. A platelet concentration of 50–0/nL in cirrhotic patients is sufficient to preserve thrombin generation at a level equivalent to the lower 10% of the normal range [18]. To investigate the effect of vWF-levels on platelet aggregation further, we measured vWF levels within a subset of cirrhotic patients as described below. Data on patient demographic and health characteristics, including age, sex, etiology of liver disease, Child-Turcotte-Pugh (CTP) score, and MELD score were obtained.

### Ethics Statement

All patients provided written informed consent, and the study was previously approved by the local ethics committee of the medical faculty of the University Hospital Heidelberg and the study was performed in accordance with the Declaration of Helsinki.

### Blood samples

Blood was drawn from the cubital or antecubital vein with a 21-gauge butterfly needle. The first milliliters of blood was collected into an EDTA-tube (ethylenediaminetetraacetic acid) and used for complete blood count. Then, citrated blood was collected for analysis of vWF. Finally, 3.8 ml of blood was collected into a tube containing 0.129 mol/l citrate (S-Monovette for PFA 100, Sarstedt AG & Co., Germany) for analysis of PFA-100 closure times.

### Modification of vWF

In patients with thrombocytopenia (platelet count <150/nL), vWF levels were modified as follows to proof the hypothesis that increased vWF can compensate for reduced platelet count in patients with liver cirrhosis: Four tubes containing 0.129 mol/l citrate (S-Monovette for PFA 100) were collected per patient. Immediately after collection, 66 µg or 165 µg of a polyclonal anti-human vWF antibody (Haematologic Technologies, Inc., U.S.A.) was added to two tubes, respectively. A third tube was prepared with 25 µg of recombinant vWF (r-vWF) (Haematologic Technologies, Inc.). We aimed to add the amount of vWF that usually is found in healthy individuals and which thus would increase vWF in healthy individuals by 100%. Based on a concentration of 10µg vWF in 1 mL plasma of healthy controls [19] and an estimated hematocrit of 35% in cirrhotic patients, 25µg r-vWF are needed to meet these assumptions. A fourth tube was used as a control. All tubes were then incubated for 60 min at 37°C in a water bath. Next, PFA-100 was performed in all samples to measure closure times using collagen-epinephrine (Col-Epi) and collagen-ADP (Col-ADP). Additionally, plasma from these same samples was used to measure vWF-antigen levels.

### Use of PFA-100 and determination of closure time

Without shaking, the samples were immediately brought to the central laboratory of the Heidelberg University Hospital, and were analyzed within 3 h after being drawn. Samples that underwent modification of vWF were analyzed immediately after incubation in a water bath.

For PFA-100 closure time analysis, a whole blood sample, collected in a tube with 3.8% sodium citrate, was used for input. It is aspirated through a capillary tube and exposed to a collagen membrane, coated with either epinephrine or ADP, at a shear rate of 5000–6000/s. Activation of platelets is caused by shear stress and exposure to the coated membrane, thus initiating the formation of a platelet plug. The time until full occlusion (and blood flow discontinuation) is measured; this is called the closure time and is a parameter for primary hemostasis [20].

PFA-100 (Siemens Healthcare, Germany) was performed using standard cartridges containing Col-Epi and Col-ADP (both Siemens Healthcare) and results for closure times were measured in seconds. Closure time >165 s with Col-Epi and that >118 s with Col-ADP were considered pathological. The test was stopped, if no clotting was achieved after 300 s; in this case, 300 s was recorded as closure time. According to recommendations and clinical practice, the PFA-100 was analyzed as following[16]: If the test with Col-Epi was prolonged, than Col-ADP was as well included for analysis. Only if this was prolonged as well, the PFA-100 was considered to be pathological.

### Analysis of vWF and ADAMTS13

vWF-antigen levels and vWF-activity were determined turbidimetric assays on a Siemens BCS XP system applying appropriate reagents (vWF Ag and INNOVANCE vWF Ac kits, respectively, all by Siemens Healthcare). Normal ranges of vWF-antigen and vWF-activity were 70–120% and 61–179%, respectively, in patients with all blood types; normal vWF-activity was 46–146% in patients with blood type O.

For measurement of ADAMTS13 activity, citrate plasma samples of 50 patients were kept in aliquots at −20°C before analysis in the central laboratory. ADAMTS13 activity was determined applying the Technozym ADAMTS-13 Activity ELISA kit (Technoclone, Heidelberg, Germany) on a Biochrom EZ Read 400 ELISA Microplate Reader at 450 nm according to the manufacturer's instructions.

### Statistical analysis

Results for closure times, vWF-antigen, vWF-activity, ADAMTS13, hematocrit, and platelet counts are reported as means with standard deviations (SD). Student's *t*-test and one way ANOVA were used to compare results of parametric data. For comparison of categorical variables, Fisher's exact test was used. Closure times, hematocrit, platelet count, vWF, and ADAMTS13 were evaluated on the basis of CTP and MELD scores. Multivariate analysis was done using a logistic regression model. Only variables showing a *P*<0.1 in univariate analysis were included for multivariate analysis. Results of the vWF-modification experiments were analyzed using the *t*-test for paired samples. Statistical analyses were performed using IBM SPSS version 21 (IBM Corp, Armonk, NY). Significance was defined as *P*<0.05. Original data can be found in Tables S1 and S2.

## Results

### Study cohort

A total of 95 patients with liver cirrhosis were screened between January 2012 and October 2013; of these, 23 were excluded because of anti-platelet drugs or NSAIDs intake. PFA-100 closure time using Col-Epi was determined in the remaining 72 patients; of these, Col-ADP was available in 62 patients.

**Figure 1:**
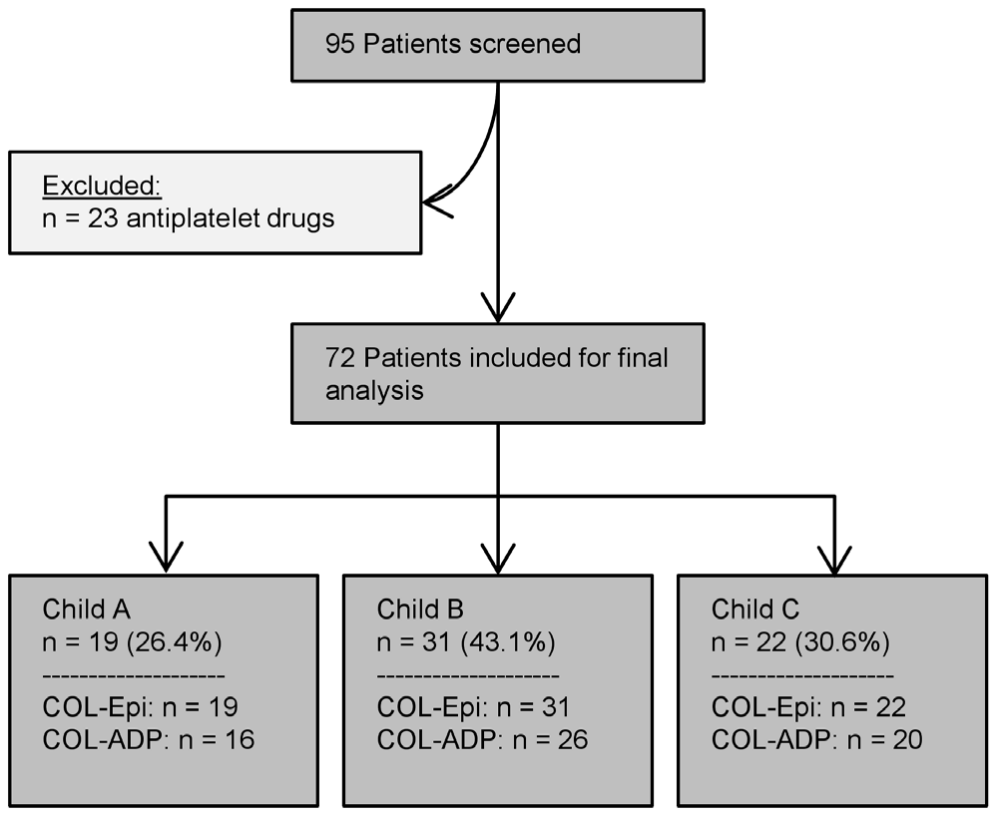
Selection of the study population. The enrolment details of the study population are shown, including the number of patients excluded because of the use of antiplatelet drugs or because there was no information on use of these drugs. For all subgroups, the number of available test results for PFA-100 with Col-Epi and Col-ADP is given.

Among the included 72 cirrhotic patients, 19 (26.4%) had Child A cirrhosis, 31 (43.1%), Child B cirrhosis, and 22 (30.6%), Child C cirrhosis; labMELD score ranged from 7 to 37 (median: 12). Further details on patient characteristics are given in Table 1. Enrolment details of the study population are shown in Figure 1.

**Table 1:** Characteristics of patients with liver cirrhosis.

	Child A	Child B	Child C	Total
Patients, n (%)	19 (26.4%)	31 (43.1%)	22 (30.6%)	72
Female, n (%)	6 (31.6%)	11 (35.5)	11 (50.0%)	28 (38.9%)
Age, yrs. (±SD)	48.5 (±8.9)	51.3 (±11.1)	52.8 (±9.4)	51.0 (±10.1)
Etiology of cirrhosis				
Cryptogenic, n (%)	1 (5.3%)	4 (12.9%)	3 (13.6%)	8 (11.1%)
Alcoholic, n (%)	8 (42.1%)	14 (45.2%)	13 (59.1%)	35 (48.6%)
Viral hepatitis, n (%)	5 (26.3%)	6 (19.4%)	5 (22.7%)	16 (22.2%)
Autoimmune hepatitis, n (%)	0	3 (9.7%)	0	3 (4.2%)
Cholestatic^a^, n (%)	3 (15.8%)	4 (12.9%)	0	7 (9.7%)
Other^b^, n (%)	2 (10.5%)	0	1 (4.5%)	3 (4.2%)
LabMELD, median (range)	8 (7–12)	12 (7–21)	19 (13–37)	12 (7–37)

Description of baseline parameters such as age, sex, and etiology of patients with liver cirrhosis and labMELD-score; grouped by Child-Turcotte-Pugh score.

a Including primary sclerosing cholangitis and biliary atresia.

b Including Wilson disease and polycystic liver disease.

### PFA-100 closure times and basic laboratory values in cirrhotic patients

The mean PFA-100 closure time with Col-Epi in cirrhotic patients was 180±62 s and that with Col-ADP was 160±70 s. Of the 72 samples tested with Col-Epi, 35 (48.6%) had results within the normal range, while 37 (51.4%) had pathological results. Of the 62 samples tested with Col-ADP, 22 (35.5%) had results within the normal range and 40 (64.5%) had pathological results. Of the patients with prolonged Col-Epi, measurement of Col-ADP was pathological in 32 (86.5%), thus a total of 32 of the 72 patients (44.4%) were classified as having a pathological test results for the PFA-100.

All patients had a mean platelet count of 109/nL ( ±63) and mean hematocrit of 34.2% ( ±6.9). Mean vWF-antigen was 383.9% ( ±200.6) mean vWF-activity was 313.5% ( ±168.7) and mean vWF-activity:vWF-antigen ratio was 0.83 ( ±0.14). Mean ADAMTS13 activity was 67.9% ( ±24.0).

**Figure 2:**
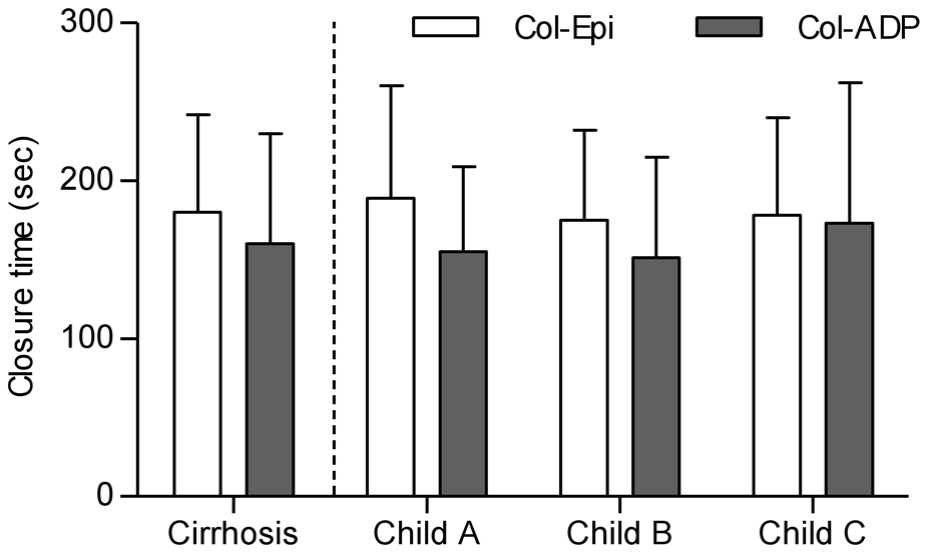
Results for PFA-100 in cirrhotic patients. Overall, mean closure times were above the upper limit of normal for Col-Epi (>165 s) and Col-ADP (>118 s) in cirrhotic patients. There were no significant differences between patients with different Child-Turcotte-Pugh scores.

Analysis by Child-Turcotte-Pugh score revealed no significant differences for mean closure times after induction with either Col-Epi or Col-ADP (Figure 2) nor in the rate of pathological PFA-100 test results. Yet there were significant differences in hematocrit, vWF-antigen and vWF-activity and a strong trend towards reduced ADAMTS13 activity in CTP B (*P* = 0.050) and CTP C (*P* = 0.070) compared to CTP A patients was observed (Table 2). Additionally, there were no significant differences in PFA-100 closure times between patients with Child-Turcotte-Pugh A, B, and C, if only patients with a platelet count ≥150/nL and hematocrit ≥27.0% were included (data not shown).

**Table 2:** Results for PFA-100, full blood count, and vWF.

	Child A	Child B	Child C	*P*<0.05
Col-Epi, n (%)	19	31	22	
Closure time, s (±SD)	189 (±71)	175 (±57)	178 (±62)	n.s.
Pathological, n (%)	10 (52.6%)	15 (48.4%)	12 (54.5%)	n.s.
Col-ADP n (%)	16	26	20	
Closure time, s (±SD)	155 (±54)	151 (±64)	173 (±89)	n.s.
Pathological, n (%)	12 (75.0%)	15 (57.7%)	13 (65.0%)	n.s.
PFA-100 test result				
Pathological, n (%)	8 (42.1%)	13 (41.9%)	11 (50.0%)	n.s.
Laboratory values				
Platelet count,/nL (±SD)	122 (±50)	115 (±80)	91 (±40)	n.s.
Hematocrit, % (±SD)	38.9 (±5.9)	34.0 (±5.9)	30.4 (±6.8)	A/B, A/C
vWF-antigen, % (±SD)	274.1 (±111.1)	358.5 (±148.6)	513.5 (±252.3)	A/C, B/C
vWF-activity, % (±SD)	223.1 (±101.7)	301.9 (±132.2)	407.3 (±212.3)	A/C
vWF-activity:vWF-antigen ratio (±SD)	0.81 (±0.11)	0.85 (±0.14)	0.83 (±0.14)	n.s.
ADAMTS13, % (SD)	81.0 (±18.9)	64.5 (±23.2)	63.7 (±26.2)	n.s.

Results for PFA-100 and basic laboratory values are grouped by Child-Turcotte-Pugh classification. The ‘*P*<0.05’-column indicates significant differences between groups according to one-way ANOVA or Fisher's exact test. For example, A/C indicates a significant difference between CTP groups A and C.

### Analysis of PFA-100 closure times in thrombocytopenic patients with liver cirrhosis

A total of 58 patients with thrombocytopenia (<150/nL) were included, among which were 29 (50.0%) with a pathological result for the PFA-100 test. This resembles a strong trend towards an increased rate of pathological results in these patients compared to the non-thrombocytopenic group (3 out of 11, *P* = 0.053). Compared to the 14 non-thrombocytopenic patients, patients with a platelet count <150/nL had prolonged closure times for Col-Epi (187±62 s vs. 149±53 s; *P* = 0.037) and Col-ADP (164±70 s vs. 140±70 s; *P* = 0.281). There were significantly more pathological results for Col-ADP (58.1% vs. 6.5%; *P* = 0.019) in the thrombocytopenic group compared to the non-thrombocytopenic patients, but this was not significant for Col-Epi (44.4% vs. 6.9%; *P* = 0.240).

Thrombocytopenic patients had significantly more advanced liver cirrhosis (median MELD 13 [range 7–37] vs. 11 [range 7–20]; *P* = 0.040 according to Mann-Whitney-*U* test), but did not show a significant difference in hematocrit (33.7±6.9% vs. 36.4±6.5%; *P* = 0.184). Levels of vWF-antigen were higher in the thrombocytopenic group (400.5±206.2% vs. 310.0±160.0%; *P* = 0.143) as were levels of vWF-activity (333.6±176.5% vs. 223.7±86.0%, *P* = 0.033).

In patients with a platelet count of less 60/nL (n = 12) closure time was 201±66 s for Col-Epi. After performing Col-ADP in the 8 (66.7%) patients with prolonged results for Col-Epi, all these patients were diagnosed with a pathological PFA-100. There was no statically significant difference in PFA closure times compared to patients with a platelet count ≥60/nL (Col-Epi: *P* = 0.203, Col-ADP: *P* = 0.341), but there was a trend towards a higher rate of pathological PFA-100 test in these patients compared to those with a platelet count ≥60/nL (*P* = 0.090).

### Analysis of vWF in thrombocytopenic patients

**Figure 3:**
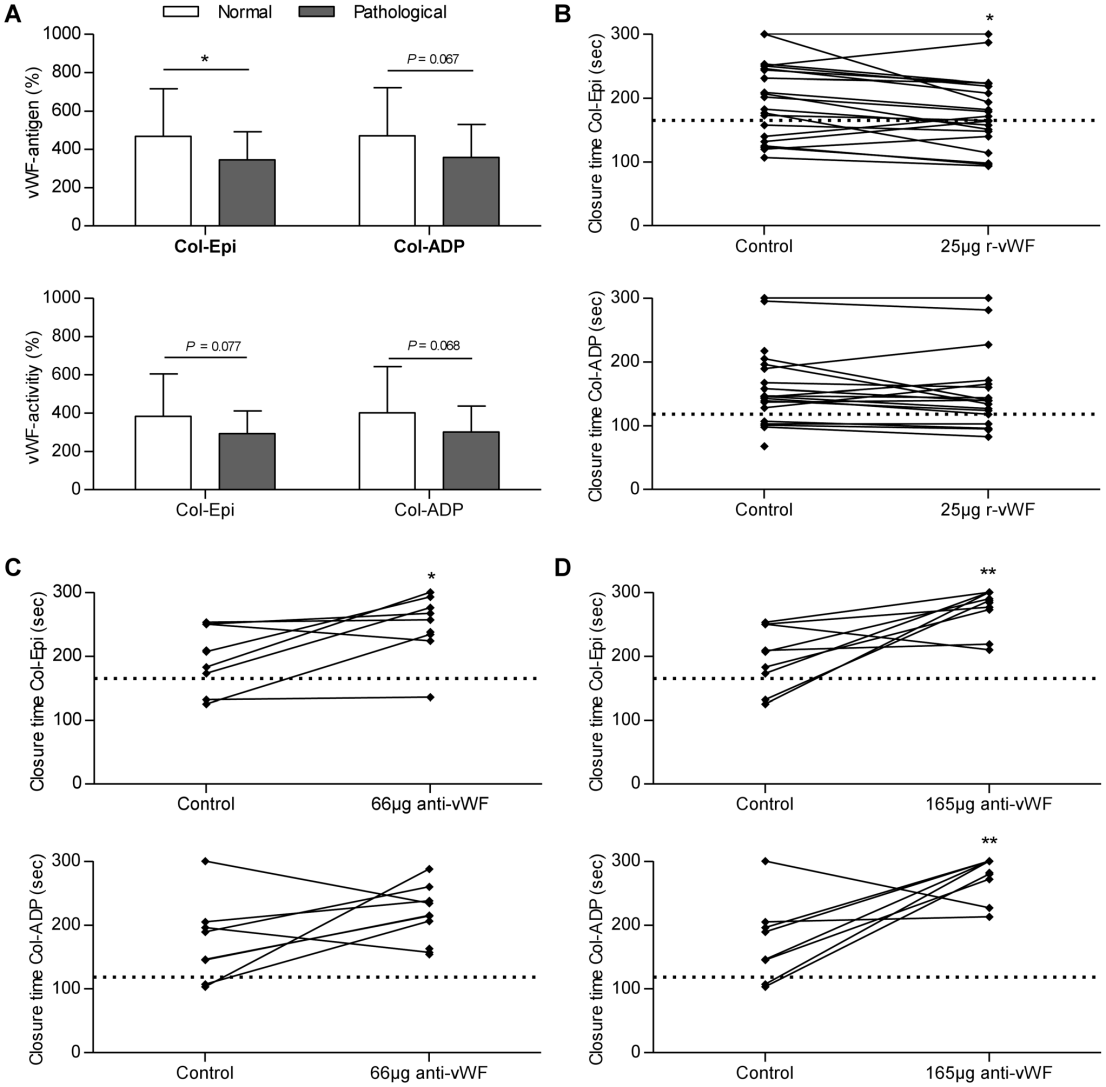
Effect of von Willebrand factor on primary hemostasis in thrombocytopenic patients with liver cirrhosis. Increased levels of vWF were associated with maintained normal primary hemostasis in patients with liver cirrhosis and reduced platelet count (<150/nL). vWF-antigen and vWF-activity were higher in patients with normal results for PFA-100 after measuring with Col-Epi and Col-ADP (A+B). Substitution of recombinant vWF (r-vWF) resulted in improved primary hemostasis as compared to unmodified samples (C). Addition of a 66-µg or 165-µg polyclonal anti-vWF-antibody levels led to prolonged closure times compared to unmodified control samples (D+E). Horizontal lines represent the upper limit of normal for closure times with Col-Epi or Col-ADP [**: *P*<0.01, *: *P*<0.05].

Analysis of vWF in thrombocytopenic patients (defined as platelet count <150/nL) revealed an association with closure times: vWF-antigen was significantly higher in those thrombocytopenic patients with a normal PFA-100 (462.3±235.9%) compared to those with pathological results (338.7±151.6%, *P* = 0.021). A similar trends was as well found for vWF-activity (376.2±209.7% vs. 291.0±125.1%, *P* = 0.065). Regarding vWF-activity:vWF-antigen ratio and ADAMTS13 activity no differences were found (*P* = 0.161 and *P* = 0.628, respectively). These results on the differences in vWF-antigen and vWF-activity were as well present when separately analyzed for Col-Epi and Col-ADP (Table 3, Figure 3A).

**Table 3:** Analysis of the effect of vWF on results of PFA-100 in thrombocytopenic patients.

	PFA-100			Col-Epi			Col-ADP		
	norm.	path.	*P*	norm.	path.	*P*	norm.	path.	*P*
vWF-antigen, % (±SD)	462.3 (±235.9)	338.7 (±151.6)	0.021	467.8 (±248.7)	345.8 (±146.3)	0.033	470.6 (±252.3)	358.3 (±171.8)	0.077
vWF-activity, % (±SD)	376.2 (±209.7)	291.0 (±125.1)	0.065	383.7 (±220.7)	292.9 (±119.1)	0.067	401.2 (±241.3)	300.9 (±135.8)	0.068
vWF-activity:vWF- antigen ratio	0.82 (±0.13)	0.87 (±0.14)	0.161	0.83 (±0.14)	0.86 (±0.14)	0.372	0.84 (±0.11)	0.86 (±0.14)	0.712

Comparison of vWF-antigen and vWF-activity in thrombocytopenic (<150/nL) patients with liver cirrhosis and the effect of vWF on results of PFA-100 measured with Col-Epi and Col-ADP (norm.  =  normal test result, path.  =  pathological test result).

### Results after modification of vWF

In 21 patients with a platelet count <150/nL, plasma vWF levels were modified with a polyclonal antibody or with recombinant vWF. Mean closure times were 197±60 s (Col-Epi) and 159±61 s (Col-ADP) in the unmodified samples. Compared to these, closure times were 178±57 s (Col-Epi; *P* = 0.019) and 149±60 s (Col-ADP; *P* = 0.189) after addition of recombinant vWF and we found a significant increase in vWF antigen levels in all 21 samples (*P*<0.001). Addition of 66 µg of anti-vWF-antibody resulted in closure times of 247±49 s for Col-Epi (*P* = 0.016) and 213±45 s for Col-ADP (*P* = 0.091); addition of 165 µg resulted in closure times of 274±33 s for Col-Epi (*P* = 0.005) and 277±32 s for Col-ADP (*P* = 0.006). Results are shown in Figure 3B-D and summarized in Table 4.

**Table 4:** Effect of vWF levels on primary hemostasis in thrombocytopenic patients with cirrhosis.

	25 **µ**g r-vWF	66 **µ**g anti-vWF	156 **µ**g anti-vWF
Col-Epi	**↓**	**↑**	**↑**
Col-ADP	**↔**	**↑**	**↑**

Overview of the main results: changes in closure times after addition of either recombinant vWF or anti-vWF-antibody (↔: no change, ↓: improved, ↑: worsened).

### Detection of thrombocytopathy in patients with normal platelet count and hematocrit

Overall, 14 patients with liver cirrhosis had a platelet count ≥150/nL and hematocrit ≥27%. Mean closures time were 149±53 s for Col-Epi. Pathological results for Col-Epi were found in 5 (35.7%) patients and after measurement of Col-ADP in these patients, a total of 3 (21.4%) patients were diagnosed with a pathological PFA-100 test. There were no differences regarding platelet count, hematocrit, or vWF between patients with normal and pathological results.

### Analysis of independent predictors of pathologic results for the PFA-100 test

The influence of the following variables on the outcome of the PFA-100 was investigated: age, sex, labMELD score, serum albumin, platelet count, hematocrit, vWF-antigen, vWF-activity:vWF-antigen ratio, and ADAMTS13. In univariate analysis only platelet count (*P* = 0.086), hematocrit (*P* = 0.054) and vWF-antigen (*P* = 0.064) levels were associated (*P*<0.1) with the outcome of the PFA-100. In a multivariate analysis including these three variables, only hematocrit (*P* = 0.027) and vWF-antigen (*P* = 0.010) turned out to significantly influence the PFA-100 test results, yet there was a strong trend for platelet count as well (*P* = 0.069). The same analysis was performed in thrombocytopenic patients (<150/nL), which revealed again hematocrit (*P* = 0.026) and vWF-antigen (*P* = 0.013) as independent predictors of the test outcome (Table 5 and 6).

**Table 5:** Results for univariate analysis of predictors of a pathological PFA-100 test result.

Variable	Exp(B)	95% KI für Exp(B)	*P*
**All patients**			
Age	0.981	0.936–1.028	0.418
Sex	0.900	0.346–2.339	0.829
MELD	1.019	0.943–1.101	0.627
Albumin	0.984	0.916–1.057	0.661
Platelet count	0.992	0.983–1.001	0.086
Hematocrit	0.930	0.864–1.001	0.054
vWF-antigen	0.997	0.994–1.000	0.064
vWF-activity:vWF-antigen ratio	13.528	0.409–447.335	0.145
ADAMTS13	0.991	0.967–1.014	0.437
**Platelets <150/nL**			
Age	0.969	0.920–1.022	0.246
Sex	1.161	0.398–3.392	0.785
MELD	0.998	0.920–1.083	0.967
Albumin	0.994	0.916–1.079	0.888
Platelet count	0.990	0.974–1.006	0.223
Hematocrit	0.932	0.860–1.010	0.086
vWF-antigen	0.996	0.993–1.000	0.032
vWF-activity:vWF-antigen ratio	17.371	0.309–967.333	0.165
ADAMTS13	0.994	0.969–1.019	0.619

Univariate analysis of the following variables was performed to identify predictors of a pathological PFA-100 test results in the whole study cohort and in thrombocytopenic cirrhotic patients. Multivariate analysis was then performed with variables showing a *P*-value ≤0.1 in univariate analysis.

**Table 6:** Results for multivariate analysis of predictors of a pathological PFA-100 test result.

Variable	Exp(B)	95% KI für Exp(B)	*P*
**All patients**			
Platelet count	0.990	0.980–1.001	0.069
Hematocrit	0.909	0.834–0.989	0.027
vWF-antigen	0.995	0.992–0.999	0.010
**Platelets <150/nL**			
Hematocrit	0.901	0.822–0.987	0.026
vWF-antigen	0.995	0.992–0.999	0.013

Variables with a P≤0.1 in univariate analysis were included in multivariate analysis, which was separately performed for the whole study cohort and for patients with a platelet count y 150/nL.

## Discussion

Over the past years, knowledge about hemostasis in patients with cirrhosis has greatly expanded and revealed that conventional coagulation parameters as International Normalized Ratio (INR), prothrombin time, or platelet count are not as reliable to indicate an increased risk of bleeding as assumed previously. Especially with regard to platelet transfusions or supplementation with coagulation factors, clinicians are in need of tests that determine the actual hemostatic balance in these patients more accurately [21]. Therefore, we conducted a study to evaluate primary hemostasis in patients with liver cirrhosis by means of PFA-100 closure times. On the one hand side our results indicate that primary hemostasis is impaired in a large number of patients and we found a high rate of results indicating thrombocytopathy. On the other hand side we also demonstrated that increased vWF levels can compensate for reduced platelet count.

PFA-100 is a validated test for screening of platelet function disorders and von Willebrand's disease [17], [22] and has been used to detect platelet-related defects of primary hemostasis in dialysis patients as well [23]. Until now, data on its use in patients with liver cirrhosis is only available from two smaller studies. The first study reported increased closure times in 20 patients with Child A or B cirrhosis. An improvement of closure times was shown after experimentally increasing hematocrit [24]. Since we found pathological results in patients with a hematocrit ≥27% in the present study, we esteem that transfusion of packed red blood cells may only improve primary hemostasis in some patients. The second study focused on differences between cholestatic and non-cholestatic causes of liver diseases. Closure times for patients with primary biliary cirrhosis and primary sclerosing cholangitis were less compared to patients with non-cholestatic causes of cirrhosis [25]. Despite including more patients, especially with end-stage liver disease, to our knowledge, this is the first study to acknowledge platelet count, hematocrit, and vWF in the analysis. The latter two were as well identified as independent predictors of maintained primary hemostasis in multivariate analysis, yet a strong trend was found for platelet count as well. Our study results support the hypothesis that cirrhosis disrupts primary hemostasis, which does seem independent on the stage of cirrhosis.

Within our samples of non-thrombocytopenic, non-anemic patients, we obtained pathological results for the PFA-100 in 21.4% of cases. Platelet dysfunction is the most likely reason for these pathological results, since patients with low hematocrit and low platelet count were not included in this analysis and vWF-antigen and vWF-activity were increased. Until now, data on the presence of thrombocytopathy in cirrhotic patients is still controversial [7]. Possible causes of thrombocytopathy in liver cirrhosis are signal transduction defects, storage pool disorders, or impaired thromboxane A2 synthesis [26], [27], [28]. According to our findings, in a system closely mimicking in-vivo primary hemostasis, platelet function defects are present in cirrhotic patients and might contribute to prolonged primary hemostasis.

Analysis of the PFA-100 test results and results for Col-Epi and Col-ADP in thrombocytopenic patients only revealed a higher rate of pathological results in some of the analysis in these patients with reduced platelet count, but results did show no differences for the other analyses. This was true for either of two cut-offs used in our analysis, namely 150/nL and 60/nL. This strengthens the hypothesis that platelet count alone as a static parameter does not resemble of the current state of coagulation in cirrhotic patients. This is further supported by a ROC (receiver operating characteristic) analysis performed to analyze whether platelet count predicts PFA-100 results. This revealed an area under the curve of 0.716 and thus showing only moderate capability of discriminating between normal and pathological PFA-100 results. The assessment of bleeding risk before medical interventions probably is not well predicted based upon a static parameter, such as platelet count [29]. Therefore tests globally and dynamically assessing primary hemostasis should be further investigated, especially since other factors, as vWF or hematocrit, might compensate for a reduced platelet count. These studies should as well evaluate if bleeding complications are related to pathological test result. Our study indicates that the PFA-100 might be useful to characterize the interplay of increased vWF and reduced platelet count in cirrhotic patients. Thus, PFA-100 should be examined in cirrhotic patients undergoing medical interventions.

In patients with cirrhosis and thrombocytopenia, maintenance of normal primary hemostasis seems to depend greatly on concomitantly increased vWF levels and reduced levels of the vWF cleaving protease ADAMTS13 [15], [30]. Evidence for this was first reported by Lisman et al. [31] and is supported by our analysis of thrombocytopenic patients and the vWF-modification experiment. In addition to the important work by Lisman et al., who used pooled plasma from healthy controls and patients with liver cirrhosis we further decided to directly modify plasma vWF levels. We were thus able to directly identify vWF as the important coagulation factor responsible for maintained primary hemostasis. We found that higher vWF-antigen and higher vWF-activity in thrombocytopenic patients were associated with normal closure times. Since there were no differences in vWF-activity:vWF-antigen ratio, we conclude that the difference is due to an increase in vWF-antigen and not due to differences in functionality of vWF. Further no influence of ADAMTS13 was noted in this setting and in the multivariate analysis performed. A decrease of ADAMTS13 in decompensated cirrhosis is in line with previous results [15]. Further, in Line with Lisman et al., we found a negative correlation between ADAMTS13 and the vWF-activity:vWF-antigen ratio (Pearson's r = −0.350, *P* = 0.013), indicating increased vWF proteolysis in some patients with consequently reduced vWF activity [31]. Yet neither ADAMTS13 nor the vWF-activity:vWF-antigen ratio were predictive of the PFA-100 test results, which was only true for vWF-antigen, which did not correlate to ADAMTS13. We thus suspect that a decrease in ADAMTS13 is not the solitary reason for increased vWF levels and thereby maintained normal primary hemostasis in cirrhotic patients. In thrombocytopenic patients, the addition of r-vWF significantly improvement closure times while addition of an anti-vWF-antibody yielded significantly prolonged closure times. Yet, it remains unclear whether there is physiological connection between increased vWF and decrease platelet count or not, but most interestingly both changes are commonly found in portal hypertension [11]. PFA-100 has previously been shown to correlate well with vWF [32]; therefore, we assume that it is an appropriate test to measure the effect of different vWF levels. Whether measurement of the PFA-100 or supplementation of vWF could be an option in clinical practice needs to be further evaluated. The test could be used to identify patients with impaired primary hemostasis who might benefit from increased platelet counts or vWF levels and to differentiate them from patients with risk of thrombosis due to use of pro-coagulant drugs. This is of importance, since the substitution of thrombopoietin is associated with increased rate of thrombosis [33] and platelet transfusion during liver transplantation is associated with increased mortality [21]. With regard to the supplementation of vWF, previous studies on the administration of desmopressin, able to induce vWF release from the endothelium, resulted in an improved in-vivo bleeding time, thereby supporting this approach [34], [35]. Yet, before this can be implemented in clinical practice further studies are needed to determine the optimal dose of r-vWF and to actually show an improvement compared to platelet transfusion in a clinical trial.

In conclusion, we found that primary hemostasis is impaired in cirrhotic patients probably because of reduced platelet count or hematocrit, but also because of thrombocytopathy. Increased vWF levels can partially compensate for reduced platelet count and the supplementation with r-vWF should further be investigated and compared with platelet transfusion.

## Supporting Information

Table S1 **Results for PFA-100 in patients with liver cirrhosis.**
(XLSX)

Table S2 **Results of the vWF modification experiment.**
(XLSX)
